# Yellow Fever Disarmed and Defied

**Published:** 1898-09

**Authors:** 


YELLOW FEVER DISARMED AND DEFIED.—A lay brother has a "sure preventive" and "cure" for the plagued thing. We give it for the benefit of all who are (now needlessly) scared. The give-away has been received at the Health Department of Texas and we have been permitted to copy it *pro bono publico:*

——————, TEXAS, May 5th, 1898.

DEAR SIR:—I beg YOU to excuse my asking for a few minutes attention, and this, because I want to tell YOU that I know a positive YELLOW FEVER PREVENTIVE which was intrusted to me by a SISTER of CHARITY SUPERINTENDENT of the great HOSPITAL of RIO JANEIRO (BRAZIL), when I was the Secretary of the FRENCH Benevolent Association. This preventive is in use by ALL Sisters of Charity, in the Hospitals of Rio De Janeiro and of CALCUTTA, India.

I was in Chattanooga, TENN, when the Yellow Fever broke out, imported by the MEMPHIS Refugees, and assisting a meeting of their DOCTORS, consulted about the fever. I told them about my preventive, which after deliberation was declared good, and accepted, and put to immediate general use by them, and by the PUBLIC. Gratuitous advertisement was given to it. From the time it was used the Yellow fever cases were gradually less numerous, and two weeks after, there was not a single patient taken to the PEST HOUSE, and the attending Doctors were perfectly IMMUNE.

This is the PREVENTIVE, which is known in CHATTANOOGA as ———'s Yellow Fever Preventive.

Take the outer rind of a LEMON, sliced in small pieces with a sharp knife, with the least PULP PEEL possible, and without any LEMON juice at all, take these small sliced pieces of the outer rind of the Lemon, and drop them in a PINT of genuine HOLLAND GIN, and after TWO hours infusion, the preventive is ready for use.

*Dose.*—One Table Spoonful early morning, before any meal, Another Spoonful during course of the day, and ONE THIRD Spoonful at bedtime.

As a rule, Any One taking this preventive will be IMMUNE. and if some get the Yellow Fever it will be in a mild form.

In consequence, in the Name of HUMANITY, and for the protection of our brave men, exposing their lives in CUBA, for the defense of our Country, I beseech YOU to take in consideration the information "Sure Yellow Fever Preventive," and have it put in immediate use.

Very respectfully yours,

A. B. ———,

30, P. C. C. K. of P.

P. S.—By my mother's side, I am a Nephew of General DILLON, who with LAFAYETTE and ROCHAMBEAU, was a Commander of the FRENCH Troops at YORKTOWN.

It will be observed that the outer peel of *one lemon* to a pint of gin (that seems to be the p'int) does the trick, and on the authority of General Dillon's nephew—mind *that*, now. Oh, these sure preventives are just bully. Let me give my personal testimony as to one of them.

In 1853, when I was a kid, the y. f. came to Vicksburg, where we were living, and prevailed as a bad epidemic. My father had fortunately gotten hold of a great secret, *how to pre-*

*vent y. f.;* how to become "immune," and he adopted it in his family. It was white mustard seed soaked in whisky. We ("uns") had to take a teaspoonful of white mustard seed reeking with whisky, three times a day, and it was religiously administered, you bet. There were four of us kids, and—would you believe it?—while everybody else in Vicksburg had yellow fever—and many persons, unblest with the knowledge of m. s. and whisky, died—all of my father's family, father, mother, self and three brothers, *had it, too*, but in so mild a form that none of us ever had it *but once, afterwards.* I had it in Galveston in 1867, and as two other doctors, Gant and Clark, attacked simultaneously with me, both died, I am persuaded that—Oh, shaw; what's the use?

---

STRAIGHT TALK:—We ask every one who reads this to turn to the department of "Abstracts and Selections" and read Dr. Emmett's editorial on the "Medical Press;"—read it, and reflect on it. The doctor tells the tale in plain language, as far as he goes. He is, no doubt, reserving for a future article a criticism of the growing evil which now obtains of *requiring* of a *journal an original article in the interest of the advertiser as a condition* to an advertising contract. Advertisers—many of them—are no longer satisfied with "reading notices;" they want *pages* of clinical reports—*by the editors*, bless you—testifying to the virtues of some proprietary article. The financial pressure thus exercised is having a ruinous effect on the medical press. If one journal refuses such contracts, there are others that will accept them; and to make one's self the conspicuous exception to a rule which has become very general is to immolate one's self on the altar of ethics and self respect. There should be concert of action on this subject by the Medical Journal Guild. Meanwhile—sink or swim, survive or perish—here's one who says *let her immolate*, for I'll be hanged if I'll write any such clinical reports.

Dr. Emmett (read first few lines of his article) overlooks the very important fact of the existence of the *Red Back*, as a "journal owned by a physician." It had been established six years when Dr. Emmett's journal came upon the stage, and has always been independent of publishers and all others.

